# An Immersive Multi-User Virtual Reality for Emergency Simulation Training: Usability Study

**DOI:** 10.2196/18822

**Published:** 2020-07-31

**Authors:** Dieter Lerner, Stefan Mohr, Jonas Schild, Martin Göring, Thomas Luiz

**Affiliations:** 1 Fraunhofer Institute for Experimental Software Engineering Research Program Digital Healthcare Kaiserslautern Germany; 2 University Hospital Heidelberg Clinic of Anesthesiology Heidelberg Germany; 3 Hannover University of Applied Sciences and Arts Interactive Reality Experiences Hannover Germany

**Keywords:** virtual reality, educational virtual realities, virtual patient simulation, virtual emergency scenario, simulation training, head-mounted display, immersive media, training effectiveness, presence experience, anaphylaxis, emergency medicine

## Abstract

**Background:**

Virtual reality (VR) is increasingly used as simulation technology in emergency medicine education and training, in particular for training nontechnical skills. Experimental studies comparing teaching and learning in VR with traditional training media often demonstrate the equivalence or even superiority regarding particular variables of learning or training effectiveness.

**Objective:**

In the EPICSAVE (Enhanced Paramedic Vocational Training with Serious Games and Virtual Environments) project, a highly immersive room-scaled multi-user 3-dimensional VR simulation environment was developed. In this feasibility study, we wanted to gain initial insights into the training effectiveness and media use factors influencing learning and training in VR.

**Methods:**

The virtual emergency scenario was anaphylaxis grade III with shock, swelling of the upper and lower respiratory tract, as well as skin symptoms in a 5-year-old girl (virtual patient) visiting an indoor family amusement park with her grandfather (virtual agent). A cross-sectional, one-group pretest and posttest design was used to evaluate the training effectiveness and quality of the training execution. The sample included 18 active emergency physicians.

**Results:**

The 18 participants rated the VR simulation training positive in terms of training effectiveness and quality of the training execution. A strong, significant correlation (r=.53, *P*=.01) between experiencing presence and assessing training effectiveness was observed. Perceived limitations in usability and a relatively high extraneous cognitive load reduced this positive effect.

**Conclusions:**

The training within the virtual simulation environment was rated as an effective educational approach. Specific media use factors appear to modulate training effectiveness (ie, improvement through “experience of presence” or reduction through perceived limitations in usability). These factors should be specific targets in the further development of this VR simulation training.

## Introduction

### Simulation Training in Emergency Medicine

Working in emergency medicine is characterized by constraints that constitute a high-risk constellation: need for situational assessment and decision making as well as initiation of appropriate emergency measures under time pressure, often under adverse external conditions and, at the same time, with little or no fault tolerance [1]. In the continuing education and training of emergency physicians, simulation therefore plays an ever-greater role than in other contexts. Usually, complex full-scale simulators with audio-video systems and extensive emergency equipment are required for the training of emergency physicians [2]. Initial studies report very high appreciation by the participants of the practical relevance, preparation for real-life emergency missions, and learning effect of this kind of training within the German Emergency Medical Services System [3].

### Virtual Reality Simulation

Virtual reality (VR) is increasingly used as simulation technology in emergency medicine, in particular for training nontechnical skills [4]. Experimental studies conducted with health care professionals, where training in VR is compared with traditional training media, often demonstrate the equivalence or even superiority of VR regarding learning or training effectiveness [5]. Highly immersive VR (ie, VR supported by VR glasses) enable spatial positioning of the users in a virtual 3-dimensional (3D) simulation environment and the feeling of being present in the VR. A high level of experiencing presence correlates positively with variables for learning or training effectiveness [6]. These immersive VR situations are capable of presenting visual information in a more realistic, 3D manner, either by providing a truly immersive experience or by presenting standardized and repeatable environments and patients that are otherwise not accessible, safe, “tangible,” or ethical. In the near future, immersive VR could be integrated regularly into existing simulation training as a complementary arrangement [7]. However, media use factors can also reduce perceived training effectiveness (eg, perceived usability) [8].

### Objectives

The goal of this feasibility study was to gain initial insights into the training effectiveness of a highly immersive multi-user VR simulation environment. Our intention was not to advocate VR simulation training as a replacement for traditional simulation training, but rather as a complement for training nontechnical skills (eg, clinical and procedural reasoning). The issues investigated in this study were: (1) How do emergency physicians assess the overall training effectiveness? and (2) How do they assess further media-specific variables influencing training in VR, such as the experience of presence, usability, cognitive load (CL), VR sickness, and intrinsic motivation?

## Methods

### Project Background

In the EPICSAVE (Enhanced Paramedic Vocational Training with Serious Games and Virtual Environments) project, a highly immersive room-scaled, multi-user, 3-dimensional simulation environment was developed for the team training of emergency medical services personnel as a complement to traditional simulation training. The higher-level learning goals were to improve clinical reasoning, procedural reasoning, and cooperation in the team [9]. Within the VR training environment, acquisition of practical skills (ie, insertion of intravenous cannula) is neither intended nor technically possible. Following early evaluation studies with future paramedics [10], the system was used with emergency physicians to validate the transfer to higher levels of expertise.

### EPICSAVE VR Simulation Environment

**Figure 1:**
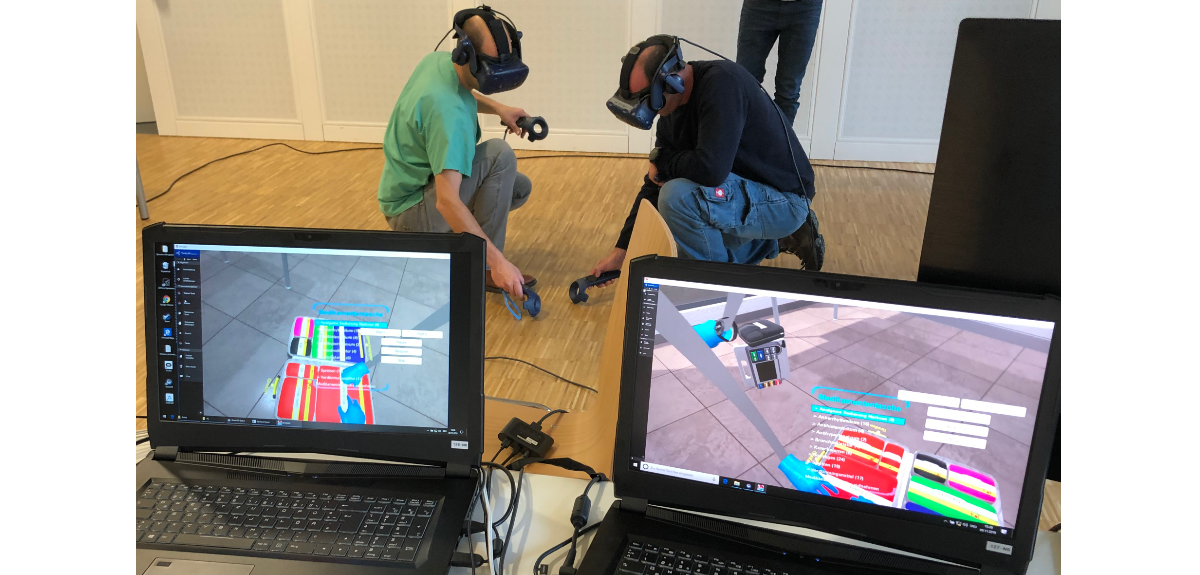
Two training participants equipped with virtual reality glasses and input devices.

For team training with 2 participants, the hardware equipment consisted of 2 sets of VR glasses with integrated headphones (head-mounted displays; HTC Vive Pro), 4 input devices (HTC Controller) for the selection of menu items or for activating and moving objects, and 2 PCs for controlling the simulation. The interactions between the training participants were transmitted to the VR in real time by means of a motion-tracking system and were executed by their virtual representatives (avatars). This enabled collaborative activities in the VR (eg, handing over virtual equipment). The trainer monitored the actions of the participants in the VR via the PC monitors (Figure 1).

An integrated virtual patient (VP) represented a multitude of vital parameters and symptom characteristics, which went beyond what can currently be represented by commercially available high-fidelity simulators (Textbox 1). Intravenous infusions or oxygen supplies emptied in real time according to the selected settings. The control of vital parameters and symptom characteristics could either be automated by the trainer using so-called presets or exercised “manually” via an easy-to-use editor. At the time the evaluation was performed, the VP was not yet equipped with language recognition and output, which is why answers from the VP were provided by the trainer off-screen.

**Symptom characteristics and measures of the virtual patient.**

**Controllable symptom characteristics of the virtual patient**

- Psychomotor condition
- Vital parameters (level of consciousness; respiratory rate, depth, and pattern; pulse rate and force; capillary refill time)
- Pathological breathing sounds
- Swelling of skin and mucous membranes
- Exanthema
- Urticaria
- Cyanosis
- Perspiration

**Diagnostic and therapeutic measures**

- Change of posture
- Undressing
- Monitor-based measurements (electrocardiogram, noninvasive automatic blood pressure, pulse oximetry, temperature, blood glucose)
- Clinical examination (inspection, palpation, auscultation)
- Administration of oxygen
- Application of infusions and medication (eg, inhalative, intravenous)
- Defibrillation

The VR system recorded the training sessions. Important diagnostic steps or therapeutic interventions were automatically recognized, displayed to the trainer in a window on their PC monitor, and presented in chronological and systematic order for the debriefing.

### Sample and Study Design

#### Sample

The 18 participating emergency physicians (14 men, 4 women) for the field study were recruited from the Clinic of Anesthesiology of the Heidelberg University Hospital following a hospital-internal written and verbal call for participation. The mean age of the participants was 36.6 years (SD 7.1 years). The mean years of practical experience was 6.4 years (SD 7.1 years). None of the participants had experience with a highly immersive VR simulation environment prior to training. They were informed about the specific emergency scenario within the training session during the briefing phase. Exclusion and discontinuation criteria for participation in the one-group pretest and posttest design study were VR sickness symptoms such as discomfort, headache, or nausea.

#### Emergency Scenario

**Figure 2:**
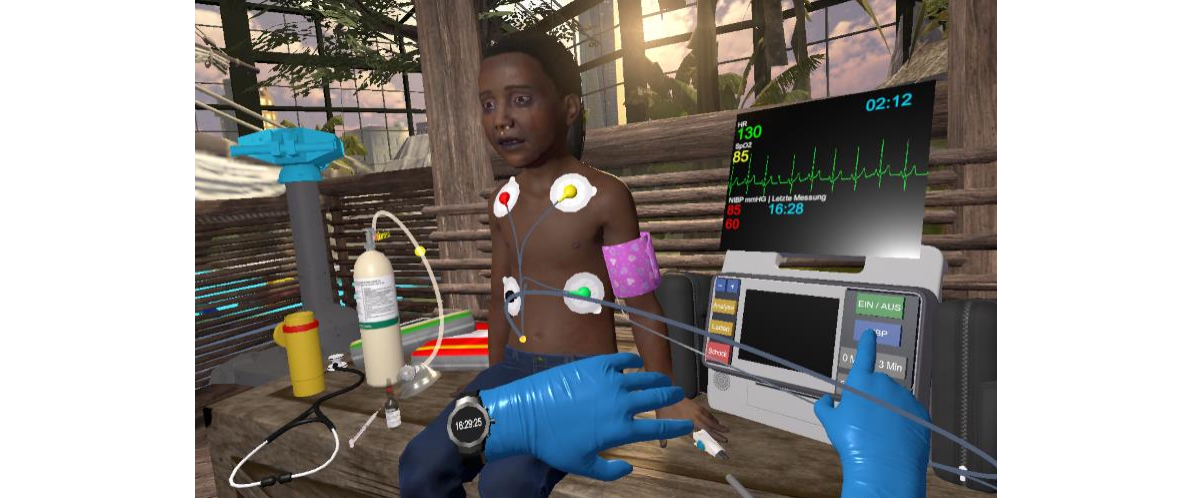
The virtual patient in the virtual environment.

The virtual emergency scenario was anaphylaxis grade III [11] with shock, swelling of the upper and lower respiratory tract, and skin symptoms in a 5-year-old girl (VP) visiting an indoor family amusement park (Figure 2). This clinical scenario was chosen because studies have shown that the potentially life-saving epinephrine therapy prioritized in the guidelines is very often carried out too late or incorrectly in practice [12,13]. Furthermore, pediatric emergencies are associated with very high levels of emotional stress for emergency medical services personnel [14].

#### Training Session

The VR training was based on the basic principle of simulation-based training [15]: briefing and VR familiarization phase (approximately 20 minutes), training (approximately 20 minutes), and debriefing (approximately 15 minutes). The team consisted of 2 physicians, while the concrete composition of the team was random. The training session was led by a VR system administrator and an experienced Advanced Life Support and Crew Resource Management Training Instructor. The central task during the training was to make a clinical decision regarding working diagnosis and therapy using the widespread Airway, Breathing, Circulation, Disability, Exposure (ABCDE) method and SAMPLER (Signs/symptoms, Allergies, Medication, Past medical history, Last meal, Events prior to incident, Risk factors) scheme while obeying the principles of effective teamwork and team communication. As part of the debriefing phase, immediate feedback for each team was given specifically on the working diagnosis, procedures for communication and teamwork, and epinephrine dose administered.

### Data Collection

#### Measurement of Training Effectiveness

Perceived training effectiveness was measured with the Training Evaluation Inventory (TEI), with the variable values ranging from 1 to 5 [16]. In addition, pretest and posttest scores of declarative and procedural knowledge were collected. The knowledge test (see Multimedia Appendix 1) consisted of 20 multiple choice questions on declarative and procedural knowledge regarding the current guidelines for prehospital diagnosis and therapy of anaphylaxis [11], with a value range of 0-20 according to the multiple-choice typology of Krebs [17]. The validity of the content was checked by 4 emergency physicians with clinical and teaching experience. A higher score corresponds to a higher level of knowledge [17].

#### Measurement of Clinical Reasoning Skills

We integrated a checklist into the VR training system as an internal embedded assessment system with the following performance data: completeness, order, and time of the ABCDE method; time until the diagnostic decision was made; time until the decision to administer adrenaline; and doses of adrenaline. Not all performance parameters could be recorded automatically due to technical problems with the VR tracking system. Therefore, some data had to be entered manually during the training. Unfortunately, we were unable to ensure that this manually entered data was precise or recorded in a timely manner. This poor reliability prevented us from using this assessment data.

#### Measurement of Additional Variables Regarding Media Use

The Igroup Presence Questionnaire (IPQ) was used to measure the experience of presence, with the variable values ranging from 0 to 6 [18]. The System Usability Scale (SUS) was used for measuring perceived usability, with the variable values ranging from 0 to 100 [19]. Perceived CL was measured with the scale by Klepsch and colleagues [20], with the variable values ranging from 0 to 7. The scale measures different aspects of cognitive load, namely intrinsic (ICL), extraneous (ECL), and germane (GCL) cognitive load. The mean values for ICL and GCL should normally be higher than those for ECL. The ECL captures the extent to which trainees engage with learning-irrelevant information during training [20]. VR sickness was assessed with a single modified item from the Simulator Sickness Questionnaire [21]. The item for assessing pretest and posttest VR sickness consisted of the question: Do you feel general discomfort (eg, symptoms such as tiredness, headache, nausea, dizziness, or difficulty with visual acuity)? The possible values were: 0 (not at all), 1 (little), 2 (perceptible), and 3 (strong). Pretest and posttest intrinsic motivation was captured with the Situational Motivation Scale (SIMS), with variable values ranging from 4 to 28, where a higher score corresponds to a higher level of intrinsic motivation [22].

Once the participants had been briefed and their consent had been obtained, the pretest was performed. Immediately after the training, the posttest was performed. For the primary data set of pretest data, see Multimedia Appendix 2; for the primary data set of posttest data, see Multimedia Appendix 3.

### Data Analysis

The data were collected in pseudonymized form and analyzed and stored in anonymized form. Bivariate and linear correlation analyses were the primarily forms of analyses. Pretest and posttest changes were assessed by means of the corresponding significance tests in accordance with the methodological literature. Regarding statistical and analysis software, SPSS (version 19) and G*Power (version 3.1.9.2) were used. The significance level for a type I error (α) was set at .05.

### Ethics Approval and Consent to Participate

The study protocol was approved by the Ethics Commission of the Medical Faculty of the University of Heidelberg, Germany (no. S-646/2018).

The participants were recruited from the Clinic of Anesthesiology of the Heidelberg University Hospital following a hospital-internal written call for participation. They were informed in writing and orally, and their written consent was obtained for the study and for publication of the study results.

## Results

### Training Effectiveness

Participant-assessed training effectiveness (TEI) resulted in a mean score of 4.38 points (SD 0.41 points). The knowledge test performed right after the conclusion of the training revealed a small, nonsignificant increase in knowledge compared to the baseline value (Table 1).

**Table 1:** Comparison of pretest and posttest knowledge test results (total score range, 0-20).

Sample	Pretest, mean (SD)	Posttest, mean (SD)	Statistical comparison			
			*t* test	*P* value^a^	*d*	beta
n=18	14.72 (1.74)	15.50 (1.77)	t_17_=1.79	.09	0.42	.50

^a^Two-sided *t* test.

### Variables of Media Use

Concerning the experience of presence (IPQ), the overall mean score was 3.79 points (SD 0.56 points). In terms of the relationship between the experience of presence (IPQ) and the TEI subscales, a significant correlation was observed mainly for the training effectiveness dimensions “Subjective enjoyment,” “Perceived usefulness,” and “Attitude towards training” (Table 2).

The bivariate correlation analysis revealed a large, significant correlation between experience of presence (IPQ) and perceived overall training effectiveness (TEI): *r*=.53, *P*=.01. In the linear regression analysis, a comparably large effect could also be seen: *R*^2^=.240, *F*_1,16_=6.368, *P*=.02, *f*=.56.

Participant-assessed usability (SUS) resulted in a mean score of 65.56 points (SD 14.54 points). In terms of the relationship between experience of presence (IPQ) and usability (SUS), a significant correlation (*r*=.44, *P*=.03) was observed.

The assessment of CL yielded the following results for the respective subdimensions: ICL (mean 3.75, SD 1.31), GCL (mean 3.74, SD 1.36), and ECL (mean 4.39, SD 1.63).

The pretest (mean 0.44, SD 0.51) and posttest (mean 0.44, SD 0.86) scores for VR sickness (modified item from the Simulator Sickness Questionnaire) were identical. Only one person scored his or her sickness as a 2 (perceptible), and one person scored his or her sickness as a 3 (strong) after the training, but neither of these participants had to stop the training because of VR sickness.

Compared to the baseline value, intrinsic motivation (SIMS) increased significantly after the training (Table 3).

The bivariate correlation analysis revealed a strong, significant correlation between experience of presence (IPQ) and situational, intrinsic motivation (SIMS) after the VR simulation training: *r*=.62, *P*=.003. In the linear regression analysis, a comparably high effect was visible: *R*^2^=.344, *F*_1,16_=9.931, *P*=.01, *f*=.72.

**Table 2:** Correlations between the Igroup Presence Questionnaire (IPQ; total score range, 0-6) and Training Evaluation Inventory (TEI) subscales (total score range, 1-5).

TEI subscales	Mean	SD	*r*	*P* value
a. Subjective enjoyment	4.67	0.40	.43	.04
b. Perceived usefulness	4.25	0.69	.52	.01
c. Perceived difficulty	4.71	0.37	.11	.34
d. Subjective knowledge gain	3.76	0.75	.39	.06
e. Attitude towards training	4.43	0.47	.54	.01

**Table 3:** Pretest and posttest results of the Situational Motivation Scale (SIMS; total score range, 4-28).

Sample	Pretest, mean (SD)	Posttest, mean (SD)	Statistical comparison			
			*t* test	*P* value^a^	*d*	beta
n=18	20.18 (4.62)	22.39 (3.42)	t_17_=2.60	.02	0.61	.52

^a^Two-sided *t* test.

## Discussion

### Principal Findings

The highly immersive virtual simulation environment enables dynamically changeable, realistic, and 3D visualization of different clinical environments and VPs from different perspectives. To get initial insights into the potential for using this VR system for simulation training, in this feasibility study, we evaluated the perceived training effectiveness and assessed further media use variables. The results regarding TEI were above average, meaning that the new VR training was not perceived as inferior.

We observed a strong, significant correlation between experience of presence and perceived training effectiveness. The values measured by the IPQ showed a rather high degree of presence experience by the training participants. A high experience of presence is highlighted in the literature as an indicator of highly interactive media, an indicator of systematic cognitive engagement with the contents of the virtual environment, and an important predictor of experience-based learning [6]. This immersion potential allows decision making and taking action without any of the disruptions or influences that may affect traditional training situations [23]. Nevertheless, the comparison between the pretest and posttest showed no significant difference regarding the results of the knowledge test, nor was there a significant correlation between the experience of presence and the posttest results for the knowledge test.

In our study, we found a strong correlation between the experience of presence and situational, intrinsic motivation. On the one hand, this may be due to high covariance caused by the novelty effect. On the other hand, a more recent study assumed a strong influence of the experience of presence on affective-emotional learning goals [8]. A comparison between the pretest and posttest results showed that VR training led to a significant increase in intrinsic motivation among the participants.

The total mean score of the SUS was 65.56 points. According to the interpretation scheme of Bangor and colleagues [24], this value corresponds to a sufficient to satisfactory rating. Some users had problems particularly with real body movement, caused by the VR headset cables. Wireless VR headsets, which have become available in the meantime, will probably reduce this problem in the future. In particular, the results for the assessment of the CL dimensions showed a higher value for ECL than for ICL or GCL. This is most likely due to usability issues, which can lead to a kind of split attention effect when trainees focus their attention on both the virtual and physical worlds [25]. Makransky and Lilleholt [8] used a structural equation model to analyze the interrelationships between VR features, presence experience, usability, learning processes, and learning results. In their analysis, they were able to show that usability influences the presence experience as well as the cognitive learning processes and therefore also has indirect and direct influences on the learning results. These findings indicate which fields should be addressed in the future to achieve overall improvement of the VR training system.

### Limitations

This feasibility study comprised a small sample of experienced emergency physicians who, by participating, expressed great interest in innovative training methods. A cross-sectional, one-group pretest-posttest design has many limitations regarding internal validity. As this VR simulation training was used only in the context of a first-time and one-time test phase, the results reported in this paper provide only a statement about this specific training duration.

A comparison of our results with other, similar studies appears problematic; due to the current variations in the configurations of VR technologies and methods used in studies, no comparable context of application could be referenced [26]. In addition, the use of subjective assessments as the primary outcome variable is associated with lower validity than the use of objective variables [27,28]. Furthermore, in future studies, validated assessment tools for nontechnical and clinical reasoning skills should be used instead of knowledge tests, which only measure declarative knowledge. These tools should take into consideration the specificity of VR simulation (eg, as a VR embedded assessment tool) [29].

Female participants were underrepresented in the sample, but this corresponds to the low percentage of women among German emergency physicians [30,31]. Bangor and colleagues [32] were able to show in an empirical study that there is no significant connection between gender and SUS scale values. Saredakis and colleagues [33] found no gender difference regarding VR sickness when using head-mounted displays. Nevertheless, in further studies, additional variables related to personality traits of the media users should be included in the analysis. The following variables should be considered: visual skills, cognitive skills such as spatial intelligence, openness towards new experiences, and immersive tendencies [34,35].

### Conclusions

As in previous evaluation studies with paramedic trainees, experienced emergency physicians assessed the virtual simulation training similarly with regard to its perceived effectiveness. In development projects where innovative educational technologies are being designed and tested, it is important to continuously measure the specific media use factors in addition to the variables for determining training effectiveness. With respect to the further development of the VR system, usability problems or technical problems should be resolved and analyzed in a more differentiated manner. Technological factors (eg, tracking, field of view, latency) that enable interactivity of the user in immersive VR have a strong negative influence on the experience of presence, CL, and performance [36-38]. In terms of research methodology, follow-up studies with control groups and a longitudinal crossover design should be initiated.

## Appendix

Multimedia Appendix 1 The knowledge test for pre- and posttest.

Multimedia Appendix 2 The primary data set of pretest data.

Multimedia Appendix 3 The primary data set of posttest data.

## Abbreviations

3D: 3-dimensional.
ABCDE: Airway, Breathing, Circulation, Disability, Exposure.
CL: cognitive load.
ECL: extraneous cognitive load.
EPICSAVE: Enhanced Paramedic Vocational Training with Serious Games and Virtual Environments.
GCL: germane cognitive load.
ICL: intrinsic cognitive load.
IPQ: Igroup Presence Questionnaire.
SAMPLER: Signs/symptoms, Allergies, Medication, Past medical history, Last meal, Events prior to incident, Risk factors.
SIMS: Situational Motivation Scale.
SUS: System Usability Scale.
TEI: Training Evaluation Inventory.
VP: virtual patient.
VR: virtual reality.

## References

[1] Urban B, Lazarovici M, Sandmeyer B, St. Pierre M, Breuer G (2018). Simulation in der Notfallmedizin- stationäre Simulation. Simulation in der Medizin: Grundlegende Konzepte - Klinische Anwendung.

[2] Marung H, Höhn M, Gräsner J, Adler J, Schlechtriemen T (2016). NASIM 25 – an option to improve emergency physician training. Notfall Rettungsmed.

[3] Armbruster W, Kubulus D, Schlechtriemen T, Adler J, Höhn M, Schmidt D, Duchêne S, Steiner P, Volk T, Wrobel M (2014). [Improvement of emergency physician education through simulator training. Consideration on the basis of the model project "NASimSaar25"]. Anaesthesist.

[4] McGrath JL, Taekman JM, Dev P, Danforth DR, Mohan D, Kman N, Crichlow A, Bond WF (2018). Using Virtual Reality Simulation Environments to Assess Competence for Emergency Medicine Learners. Acad Emerg Med.

[5] Kyaw BM, Saxena N, Posadzki P, Vseteckova J, Nikolaou CK, George PP, Divakar U, Masiello I, Kononowicz AA, Zary N, Tudor Car L (2019). Virtual Reality for Health Professions Education: Systematic Review and Meta-Analysis by the Digital Health Education Collaboration. J Med Internet Res.

[6] Mantovani F, Castelnuovo G, Riva G, Davide F, Ijsselsteijn W (2003). Sense of Presence in Virtual Training: Enhancing Skills Acquisition and Transfer of Knowledge through Learning Experience in Virtual Environments. Being There - Concepts, Effects And Measurements Of User Presence In Synthetic Environments.

[7] Radianti J, Majchrzak TA, Fromm J, Wohlgenannt I (2020). A systematic review of immersive virtual reality applications for higher education: Design elements, lessons learned, and research agenda. Computers & Education.

[8] Makransky G, Lilleholt L (2018). A structural equation modeling investigation of the emotional value of immersive virtual reality in education. Education Tech Research Dev.

[9] Kononowicz AA, Zary N, Edelbring S, Corral J, Hege I (2015). Virtual patients--what are we talking about? A framework to classify the meanings of the term in healthcare education. BMC Med Educ.

[10] Lerner D, Pranghofer J, Franke A (2020). The impact of presence experience on learning and training effects in a virtual reality simulation environment. Pädagogik der Gesundheitsberufe.

[11] Ring J, Beyer K, Biedermann T, Bircher A, Duda D, Fischer J, Friedrichs F, Fuchs T, Gieler U, Jakob T, Klimek L, Lange L, Merk HF, Niggemann B, Pfaar O, Przybilla B, Ruëff Franziska, Rietschel E, Schnadt S, Seifert R, Sitter H, Varga E, Worm M, Brockow K (2014). Guideline for acute therapy and management of anaphylaxis: S2 Guideline of the German Society for Allergology and Clinical Immunology (DGAKI), the Association of German Allergologists (AeDA), the Society of Pediatric Allergy and Environmental Medicine (GPA), the German Academy of Allergology and Environmental Medicine (DAAU), the German Professional Association of Pediatricians (BVKJ), the Austrian Society for Allergology and Immunology (ÖGAI), the Swiss Society for Allergy and Immunology (SGAI), the German Society of Anaesthesiology and Intensive Care Medicine (DGAI), the German Society of Pharmacology (DGP), the German Society for Psychosomatic Medicine (DGPM), the German Working Group of Anaphylaxis Training and Education (AGATE) and the patient organization German Allergy and Asthma Association (DAAB). Allergo J Int.

[12] Sclar DA, Lieberman PL (2014). Anaphylaxis: underdiagnosed, underreported, and undertreated. Am J Med.

[13] Carrillo E, Hern HG, Barger J (2016). Prehospital Administration of Epinephrine in Pediatric Anaphylaxis. Prehosp Emerg Care.

[14] Bernhard M, Aul A, Helm M, Mutzbauer T, Kirsch J, Brenner T, Hainer C, Gries A (2008). Invasive Notfalltechniken in der Notfallmedizin. Notfall Rettungsmed.

[15] Gaba DM (2004). The future vision of simulation in health care. Qual Saf Health Care.

[16] Ritzmann S, Hagemann V, Kluge A (2013). The Training Evaluation Inventory (TEI) - Evaluation of Training Design and Measurement of Training Outcomes for Predicting Training Success. Vocations and Learning.

[17] Krebs R (2014). Instructions for the Development of Multiple-Choice Questions and Tests in Medical Education. https://www.iml.unibe.ch/themen/uebersichten/artikel/wie-mc-fragen-pruefungsreif-werden.

[18] Schubert T, Friedmann F, Regenbrecht H (2001). The Experience of Presence: Factor Analytic Insights. Presence: Teleoperators and Virtual Environments.

[19] Brooke J, Jordan PW, Thomas B, McClelland IL, Weerdmeester B (1996). SUS. Usability evaluation in industry.

[20] Klepsch M, Schmitz F, Seufert T (2017). Development and Validation of Two Instruments Measuring Intrinsic, Extraneous, and Germane Cognitive Load. Front Psychol.

[21] Kennedy RS, Lane NE, Berbaum KS, Lilienthal MG (1993). Simulator Sickness Questionnaire: An Enhanced Method for Quantifying Simulator Sickness. The International Journal of Aviation Psychology.

[22] Guay F, Vallerand RJ, Blanchard C (2000). On the Assessment of Situational Intrinsic and Extrinsic Motivation: The Situational Motivation Scale (SIMS). Motivation and Emotion.

[23] Winn W, Jackson R (1999). Fourteen Propositions about Educational uses of Virtual Reality. Educational Technology.

[24] Bangor A, Kortum P, Miller J (2009). Determining what individual SUS scores mean: Adding an adjective rating scale. Journal of Usability Studies.

[25] van der Land S, Schouten AP, Feldberg F, van den Hooff B, Huysman M (2013). Lost in space? Cognitive fit and cognitive load in 3D virtual environments. Computers in Human Behavior.

[26] Jensen L, Konradsen F (2017). A review of the use of virtual reality head-mounted displays in education and training. Educ Inf Technol.

[27] von Wendt CEA, Niemi-Murola L (2018). Simulation in Interprofessional Clinical Education: Exploring Validated Nontechnical Skills Measurement Tools. Simul Healthc.

[28] Chauvin A, Truchot J, Bafeta A, Pateron D, Plaisance P, Yordanov Y (2018). Randomized controlled trials of simulation-based interventions in Emergency Medicine: a methodological review. Intern Emerg Med.

[29] Bracq M, Michinov E, Jannin P (2019). Virtual Reality Simulation in Nontechnical Skills Training for Healthcare Professionals: A Systematic Review. Simul Healthc.

[30] Luiz T, Jung J, Flick S (2014). [Quo vadis, preclinical emergency physician?: results of a survey of emergency medical services in Rhineland-Palatinate (Germany)]. Anaesthesist.

[31] Ilper H, Kunz T, Walcher F, Zacharowski K, Byhahn C (2013). [An online emergency physician survey - demography, education and experience of German emergency physicians]. Dtsch Med Wochenschr.

[32] Bangor A, Kortum PT, Miller JT (2008). An Empirical Evaluation of the System Usability Scale. International Journal of Human-Computer Interaction.

[33] Saredakis D, Szpak A, Birckhead B, Keage HAD, Rizzo A, Loetscher T (2020). Factors Associated With Virtual Reality Sickness in Head-Mounted Displays: A Systematic Review and Meta-Analysis. Front Hum Neurosci.

[34] Wallach HS, Safir MP, Samana R (2009). Personality variables and presence. Virtual Reality.

[35] Ling Y, Nefs HT, Brinkman W, Qu C, Heynderickx I (2013). The relationship between individual characteristics and experienced presence. Computers in Human Behavior.

[36] Cummings JJ, Bailenson JN (2016). How Immersive Is Enough? A Meta-Analysis of the Effect of Immersive Technology on User Presence. Media Psychology.

[37] Wilson ML, Beadle SC, Kinsella AJ, Mattfeld RS, Hoover AW, Muth ER (2020). Task performance in a head-mounted display: The impacts of varying latency. Displays.

[38] Frederiksen JG, Sørensen Stine Maya Dreier, Konge L, Svendsen MBS, Nobel-Jørgensen Morten, Bjerrum F, Andersen SAW (2020). Cognitive load and performance in immersive virtual reality versus conventional virtual reality simulation training of laparoscopic surgery: a randomized trial. Surg Endosc.

