# Supplementary material for: An Immersive Multi-User Virtual Reality for Emergency Simulation Training: Usability Study
Source: JMIR Serious Games. 2020 Jul 31;8(3):e18822. doi: 10.2196/18822 (PMC7428918; doi:10.2196/18822)
Supplement: Multimedia Appendix 1 [file games_v8i3e18822_app1.docx]

The **knowledge test** used (see below) consisted of 20 multiple-choice questions (originally in German) on declarative and procedural knowledge regarding the respective emergency scenario. The test was created using the relevant specialist literature. The question and answer formats were adapted to the MC typology of Krebs (2004) and presented to four emergency physicians for checking the content validity (Krebs 2004). The following table provides an overview of the medical literature used. (Lerner/Mohr/Göring/Luiz)

Tabelle : Medical literature used for the knowledge tests

| Topics of the emergency scenario | | Literature | |  |
| --- | --- | --- | --- | --- |
| Anaphylaxis | | - Hoedtke & Wirtz 2016 - Ring et al. 2014 - Simons et al. 2015 | |  |
| Emergency care | | - Bürkle 2017 | |  |
| Working according to guidelines | | - Semmel 2016 | |  |
|  | | | |  |
| Question 1-Type A positive | Answer | | Choice | |
| What is the most common cause of severe anaphylactic reactions in children? | drug | |  | |
|  | insecticide | |  | |
|  | food | | x | |
|  | latex  preservative | |  | |
|  |  |  |  | |
| Question 2-Type A positive | Answer | | Choice | |
| What is the most common cause of severe anaphylactic reactions in adults? | drug | |  | |
|  | insecticide | | x | |
|  | food | |  | |
|  | latex  preservative | |  | |
|  |  |  |  | |
| Question 3-Type A positive | Answer | | Choice | |
| Which food most often triggers anaphylaxis in children and adolescents (0-17 years)? | fish | |  | |
|  | peanut | | x | |
|  | wheat flour | |  | |
|  | shellfishes | |  | |
|  | cow`s milk | |  | |
| Question 4 k-prim | Answer | | Choice | |
| The regular use of which medication (pre-medication) does not usually lead to an exacerbation of the anaphylactic symptoms? | ACE inhibitor | |  | |
|  | Beta blocking agents | |  | |
|  | Prednisolone | | x | |
|  | Non-steroidal anti-inflammatory drugs (NSAR) | |  | |

| Question 5 k-prim | Answer | Choice |
| --- | --- | --- |
| Which organ systems can manifest anaphylactic reactions? | On the skin | x |
|  | On the airways | x |
|  | On the gastrointestinal tract | x |
|  | On the cardiovascular | x |
| Question 6 k-prim | Answer | Choice |
| Which of the following statements about clinical symptoms are correct? | The symptoms of an anaphylactic reaction can occur in different ways at the same time or one after the other. | x |
|  | There are always a) cutaneous reactions before b) pulmonary and c) cardiovascular reactions. |  |
|  | Circulatory reactions can primarily occur without prior cutaneous or pulmonary reactions. | x |
|  | The anaphylactic reaction can stop spontaneously at any stage. | x |
| Question 7 k-prim | Answer | Choice |
| Which of the following statement(s) about the manifestation of the clinical symptoms of anaphylaxis on the skin and mucous membranes is / are correct? | Itching, erythema, urticaria and angioedema only show up on areas of the skin that have had direct contact with the trigger (e.g. insect bite). |  |
|  | The skin is most commonly affected by anaphylaxis. | x |
|  | At the beginning of anaphylaxis, itching or burning sensation on the palms of the hands and soles of the feet may be noticeable in the sense of prodromal symptoms. | x |
|  | Swelling of the tongue, hypopharynx, or larynx can quickly become life-threatening. | x |
| Question 8-Type A negative | Answer | Choice |
| Which of the diagnoses listed does not represent a meaningful differential diagnosis of anaphylaxis? | Vasovagal syncope |  |
|  | alcohol intoxication |  |
|  | primary tuberculosis | x |
|  | Thyrotoxic crisis |  |
|  | Angioneurotic edema |  |
| Question 9-Type A positive | Answer | Choice |
| Which patient group is at increased risk of dying from anaphylaxis? | Patients with a recent stroke |  |
|  | asthmatic | X |
|  | Patients on anticoagulants |  |
|  | Patients with acute psychosis |  |
|  | Children aged 3 - 6 years |  |
| Question 10-Type A positive | Answer | Choice |
| Which application procedure is not recommended for anaphylaxis? | i.m. (intramuscularly) |  |
|  | i.v. (intravenously) |  |
|  | s.c. (subcutaneously) | X |
|  | i.o. (intraosseous) |  |
|  | p.i. (by inhalation) |  |

| Question 11-Type A positive | Answer | Choice |
| --- | --- | --- |
| What dosage is recommended for intramuscular adrenaline administration in non-resuscitated patients? | 0,001 mg/kg BW |  |
|  | 0,01 mg/kg BW | X |
|  | 0,05 mg/kg BW |  |
|  | 0,1 mg/kg BW |  |
|  | 1 mg/kg BW |  |
| Question 12-Type A positive | Answer | Choice |
| Which adrenaline auto-injector do you use for a young patient who is not subject to resuscitation (18 years, 70 kg)? | Adrenalin-Autoinjector 150 µg |  |
|  | Adrenalin-Autoinjector 300 µg |  |
|  | Adrenalin-Autoinjector 500 µg | x |
|  | Adrenalin-Autoinjector 800 µg |  |
|  | Adrenalin-Autoinjector 1000µg |  |
| Question 13-Type A positiv-Frage) | Answer | Choice |
| Where is the optimal application site for i.m. adrenaline administration in the context of anaphylaxis? | upper outer third of the thigh |  |
|  | middle outer third of the thigh | X |
|  | lower outer third of the thigh |  |
|  | upper inner third of the thigh |  |
|  | lower inner third of the thigh |  |
| Question 14-Type A positive | Answer | Choice |
| You will meet a 4-year-old young child with 20 kg body weight and a massive asthma attack (massive cyanosis, intercostal retraction, severe restlessness) and intestinal cramps after enjoying ice cream with peanuts. Which drug therapy do you take first? | Salbutamol 2,5 mg inhalator |  |
|  | Prednison 100 mg Suppos. rectal |  |
|  | Adrenalin 0,2 mg i. m. injection | x |
|  | Prednison 10mg i. v. injection |  |
|  | Fenistil 2mg i. v. injection |  |
| Question 15-Type A positive | Answer | Choice |
| How many liters of oxygen should be used by which route of administration in the case of a severe anaphylactic reaction from grade II? | between 2-3 l / min oxygen are administered through a mask. |  |
|  | between 6-8 l / min oxygen are administered through nasal cannula. |  |
|  | 10-15 l / min oxygen can be administered through a mask. | x |
|  | between 2-3l / min oxygen through nasal cannula |  |
|  | 10-15 l / min oxygen through nasal cannula |  |
| Question 16 k-prim | Answer | Choice |
| Which of the following statements about volume therapy for anaphylaxis is correct?  Please check every statement. | Artificial colloidal solutions (e.g. HES 6% 200 / 0.5 or gelatin) should always be given. |  |
|  | Balanced full electrolyte solutions should always be given first. | x |
|  | Primarily, natural colloidal solutions (e.g. human albumin) should always be given. |  |
|  | NaCl injection solutions should always be given first |  |

| Question 17-Special format order, evaluation like k-prim | Answer | Order |
| --- | --- | --- |
| You arrive at an emergency patient.  How do you basically proceed with the primary assessment?  Put the following steps in the correct order by numbering the answer formats from 1 to 5. | Check: cyanosis, tachypnea, SpO2 drop? | 2 |
|  | Check: e.g. B. Skin changes | 5 |
|  | Check: respiratory swelling, hoarseness, stridor? | 1 |
|  | Check: confusion, loss of consciousness? | 4 |
|  | Check: paleness, cold sweat, hypotension, tachycardia, extended recapillarization time? | 3 |
| Question 18-Type A positive | Answer | Choice |
| At what grade of anaphylaxis do you generally administer adrenaline (i.m.) to children or adults? | Already from the history of known allergies? |  |
|  | from Grade I |  |
|  | from Grade II | X |
|  | from Grade III |  |
|  | from Grade IV |  |
| Question 19-Type A positive | Answer | Choice |
| You will meet a 25-year-old patient with the following symptoms: hoarseness, mild dyspnea, flush, angioedema, tachycardia 125 / min, blood pressure 95/60 mmHg.  What grade of an anaphylactic reaction is it? | Grade I |  |
|  | Grade II | x |
|  | Grade III |  |
|  | Grade IV |  |
|  | Grade V |  |
| Question 20-Type A positive | Answer | Choice |
| You meet a 25-year-old patient with the following symptoms: bronchospasm, flush, angioedema, tachycardia 145 / min, blood pressure 65/35 mmHg.  What grade of an anaphylactic reaction is it? | Grade I |  |
|  | Grade II |  |
|  | Grade III | x |
|  | Grade IV |  |
|  | Grade V |  |
